# Tailed bacteriophages (Caudoviricetes) dominate the microbiome of a diseased stingless bee

**DOI:** 10.1590/1678-4685-GMB-2023-0120

**Published:** 2024-01-19

**Authors:** Lilian Caesar, Karen Luisa Haag

**Affiliations:** 1 Indiana University Bloomington, Department of Biology, Bloomington, IN, USA.; 2 Universidade Federal do Rio Grande do Sul, Departamento de Genética, Programa de Pós-Graduação em Genética e Biologia Molecular, Porto Alegre, RS, Brazil.

**Keywords:** Metagenome, stingless bee, bacteriophage, virus, disease

## Abstract

Bacteriophages, viruses that infect bacterial hosts, are known to rule the dynamics and diversity of bacterial populations in a number of ecosystems. Bacterial communities residing in the gut of animals, known as the gut microbiome, have revolutionized our understanding of many diseases. However, the gut phageome, while of apparent importance in this context, remains an underexplored area of research. Here we identify for the first time genomic sequences from tailed viruses (Caudoviricetes) that are associated with the microbiome of stingless bees (*Melipona quadrifasciata*). Both DNA and RNA were extracted from virus particles isolated from healthy and diseased forager bees, the latter showing symptoms from an annual syndrome that only affects *M. quadrifasciata*. Viral contigs from previously sequenced metagenomes of healthy and diseased forager bees were used for the analyses. Using conserved proteins deduced from their genomes, we found that Caudoviricetes were only present in the worker bee gut microbiome from diseased stingless bees. The most abundant phages are phylogenetically related to phages that infect Gram-positive bacteria from the order Lactobacillales and Gram-negative bacteria from the genus *Gilliamella* and *Bartonella*, that are common honey bee symbionts. The potential implication of these viruses in the *M. quadrifasciata* syndrome is discussed.

Metagenomics revealed that viruses are the most abundant biological entities on Earth (Edwards and Rohwer 2005). Most of these viruses are bacteriophages, predators of bacteria, and archaeal viruses. It is estimated that every 48 hours half of all bacteria on Earth are killed by them (Shkoporov *et al*., 2022). Bacteriophages have fundamental roles in niches as distinct as the oceans’ sediments, where they participate in key biogeochemical processes, such as nutrient cycling (Fuhrman, 1999), and the gut microbiome, where they control bacterial densities (Townsend *et al*., 2021). In the human gut, phages interact directly with their prey, and indirectly with the human immune system, being major players in human health and disease (Seo and Kweon, 2019). Honey bees are models for the study of microbiomes (Zheng *et al*., 2018) and their gut ‘phageome’ has been recently characterized focusing on the diversity, host range and functional potential of bacteriophages (Bonilla-Rosso *et al*., 2020; Deboutte *et al*., 2020; Busby *et al*., 2022). It was found that most phages in the honey bee gut are virulent (Busby *et al*., 2022), viruses that do not insert themselves in bacterial genomes in the form of prophages, but instead hijack their host cells and use their resources to make new phages, causing the cell to lyse and die in the process. There is clear evidence that not all bacteria are infected by all phages, and that most phages can only infect a subset of bacterial species, *i.e.,* they show host specificity (Koskella and Meaden, 2013). Thus, phages are modulators of the gut microbiota, which is essential for bee development, pollen digestion and immunity (Deboutte *et al*., 2020).

In recent years we have been investigating the composition and dynamics of the bacterial and fungal gut communities of a stingless bee, *Melipona quadrifasciata* Lepeletier 1836. Some microbiome changes are associated with an annual syndrome that often leads the colony to collapse (Díaz *et al*., 2017; Haag *et al*., 2023). We also used metagenomics to identify differences in the virome composition of healthy and diseased stingless bees. Our data allowed us to characterize seven novel viruses with the potential of causing the neurological symptoms that we observed in some of the diseased colonies, but none of them was consistently associated with the disease outbreaks (Caesar *et al*., 2019). Gene expression studies on diseased *vs.* healthy *M. quadrifasciata*, rather suggest that the underlying causes of the annual syndrome are multifactorial, and involve a weakening process of the bee colony that culminates in March when outbreaks occur synchronously in different regions of southern Brazil (Caesar *et al*., 2022). Between January and March forager bees lose weight and bacterial counts increase in the gut, suggesting a relaxation in the bee immunologic mechanisms that regulate bacterial growth (Haag *et al*., 2023). Here we use the metagenomic data generated in our previous virome characterization (Caesar *et al*., 2019) to identify bacteriophages that may participate in the process of modulating the *M. quadrifasciata* gut microbiota. 

Our analyses were run on four assemblies built from sequences generated by high throughput sequencing of virus particles separated by centrifugation (Caesar *et al*., 2019; BioProject PRJNA960650). Two viral contig assemblies (DNA and RNA) came from a single pooled sample of worker bees from a diseased hive of *M. quadrifasciata*. The other two assemblies (DNA and RNA) are from a healthy hive of *M. quadrifasciata* sampled simultaneously at the same meliponary. For the identification of bacteriophage contigs we used the four sets as inputs on two softwares, geNomad (Camargo *et al*., 2023) and VirSorter2 (Guo *et al*., 2021). GeNomad was run end-to-end, using the flags *--cleanup --splits* 8 and the default virus score cutoff of >0.7. VirSorter2 was run with the flags *--min-score 0.5 --keep-original-seq --hallmark-required-on-short*. To confirm the identification of phages and check the quality of metagenome-assembled genomes (MAGs) we used CheckV (Nayfach *et al*., 2021) with default commands. All identified phage contigs were retained for the following analyses since they had 0 host genes, and the majority of them had at least 1 viral hallmark gene.

Using both geNomad and VirSorter a total of 193 viral contigs were identified as phages, and went through the downstream analyses. Within the four assemblies, bacteriophages were only found among the unhealthy DNA and RNA contigs (n=64 and 129, respectively; Table 1). GeNomad also performs gene prediction with a modified version of Prodigal (Hyatt *et al*., 2010) called prodigal-gv, and assigns the predicted proteins to geNomad’s markers using MMseqs2 (Steinegger and Söding, 2017). The vast majority of the annotations of predicted genes from both datasets (DNA and RNA) were assigned to bacteriophage markers belonging to the Caudoviricetes (Tables 1 and S1), a class of virulent phages known as the tailed phages, which currently contains the majority of the total phage sequences in public databases (Zhu *et al*., 2022).

**Table 1 -:** Phage distribution, taxonomy and abundance among the four metagenomes analyzed in the present study.

	Healthy		Diseased	
	DNA	RNA	DNA	RNA
Total number of viral contigs^a^	3	15	343	1559
Number of phage contigs	0	0	64	129
Number of vOTUs	0	0	56	127
Phage taxonomy	-	-	Caudoviricetes	Caudoviricetes
Phage vOTU completeness	-	-	0.2 - 47.95 %	0.11 - 45.29 %
Phage vOTU coverage	-	-	0.05 - 2,704.15 X	1.28 - 3,134.75 X

^a^ Caesar *et al*., 2019.

Next, contigs on each sample were de-replicated with dRep (Olm *et al*., 2017), using flags *--P_ani 0.95 --cov_thresh 0.85*, and coverage estimated by mapping the original reads against them with Bowtie2 (Langmead and Salzberg 2012), using the flag *--no-discordant* and *--very-sensitive.* The database for mapping reads included also non-phage contigs from the assembly to avoid non-specific or low quality reads mapping against the phage sequences. Coverage bam files were used as inputs along with the contigs for binning the phage genomes with vRhyme, using default commands (Kieft *et al*., 2022). Binned and non-binned sequences from both samples were then de-replicated with dRep using the same parameters that follow the standard thresholds for vOTU classification (Roux *et al*., 2019). Coverage of vOTUs in each sample was recovered again with Bowtie2 and command *depth -a* from Samtools (Li *et al*., 2009). Coverage and completeness estimates for the 182 vOTUs from both assemblies (DNA and RNA) are listed in Table S2. Coverage estimates ranged between 0.05 - 2704.15X (DNA) and 1.28 - 3134.75X (RNA). Completeness varied between 0.2 - 47.95% and 0.11 - 45.29%, for vOTUs from the DNA and RNA assemblies, respectively (Table 1). Thus, with the sequencing effort employed in our study, we assembled incomplete genomes, probably from the most abundant bacteriophages found in the gut of our diseased stingless bees (Figure 1).

**Figure 1 -:**
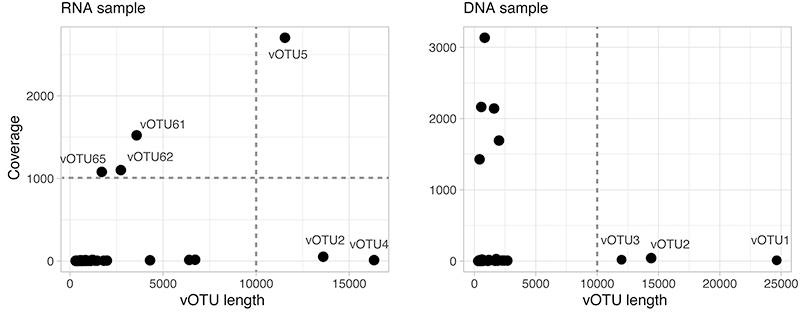
Coverage estimates for vOTUs of variable lengths (bp) assembled from the unhealthy *M. quadrifasciata* DNA and RNA samples. Each dot on the chart corresponds to a phage MAG. Horizontal and vertical dashed lines mark the coverage and length thresholds, respectively, to keep vOTUs for the downstream analyses (see text for details).

We attempted to predict phage taxa and their putative hosts based on MAGs similarity to known phages, and on their match to CRISPR-spacers. First, proteins encoded by all phage vOTUs were predicted and primarily annotated with Prokka (Seemann, 2014), using flag *-- kingdom Viruses.* Proteins and contigs from the 5 largest vOTUs were used together with previously described bee phage sequences (Bonilla-Rosso *et al*., 2020; Deboutte *et al*., 2020; Busby *et al*., 2022) as inputs for vConTACT2 (Bin Jang *et al*., 2019). The program vConTACT2 was ran with default commands, using the ‘ProkaryoticViralRefSeq211-Merged’ database, and results were visualized with the R package Igraph. Only vOTU1 and vOTU3 could be connected to previously described phages, but with low confidence and none of the vOTUs clustered with any phage (Figure 2). Additionally, we used all phage contigs as queries in BLASTn, using flags *-evalue 1e-3 -ungapped -perc_identity 95,* against three CRISPR-spacer databases: CrisprOpenDB (Dion *et al*., 2021), spacers from honey bee microbiome-associated bacteria (Bonilla-Rosso *et al*., 2020), and spacers from stingless bees’ microbiome-associated bacteria. The latter database was built based on spacers that we predicted in complete genomes of bacteria sequenced from six species of stingless bees (Sarton-Lohéac *et al*., 2023) using the ‘CRISPRCasFinder’ online tool, with default settings, and evidence level 3 or 4. Unfortunately, none of our vOTUs have matches with CRISPR spacers from any of the three searched databases. We think that traditional approaches to identify viral hosts that are based on genomic data from bacteria and their phages are of limited use in the context of our study, since there are so far only a few characterized genomes from stingless bee microbiomes (*e.g*. Sarton-Lohéac *et al*., 2023). 

**Figure 2 -:**
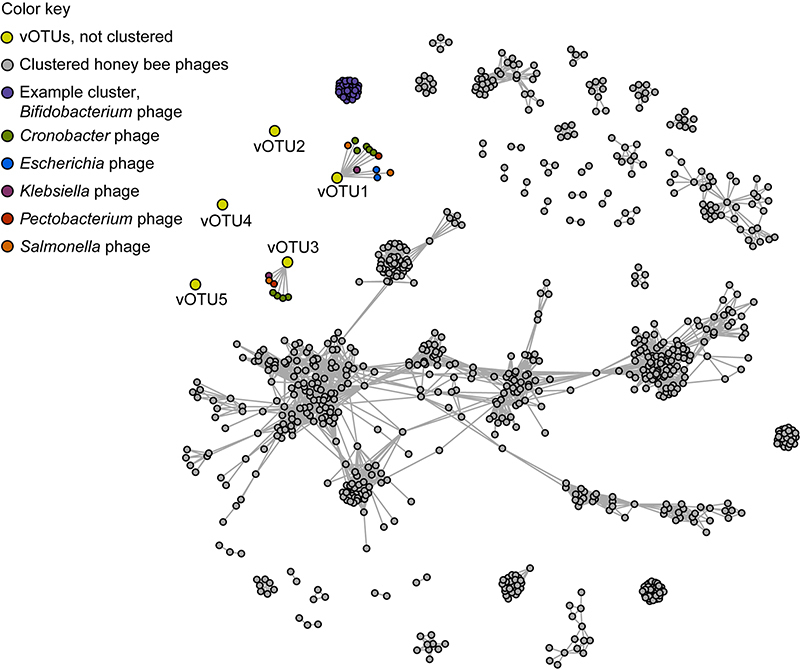
Protein-sharing network displaying unclustered *M. quadrifasciata* phages and clustered phages of *Apis mellifera*. Nodes represent phage partial to complete genomes, and edges connecting them indicate a statistically significant similar protein profile between their genomes. No vOTU >10 kb clustered with known phages in the database, and only two had weak connections with known phages.

In a further attempt to infer the putative bacteria used as hosts by the dominant tailed phages inferred from our study (vOTUs 2, 5, 61, 62 and 65; see Figure 1 and Table S2) we used phylogenetic analyses. These MAGs showed the highest coverage in the RNA metagenome, which we interpreted as a proxy for their abundance in the unhealthy bee microbiome. Moreover, these MAGs contain hallmark genes that could be used for BLAST searches of related sequences. For vOTUs 2, 5 and 65 we used a gene encoding the DNA encapsidation protein (terminase) as phylogenetic marker. The protein deduced from vOTUs 5 and 65 was identical. For vOTUs 61 and 62 we used the predicted major capsid protein, which was also identical in both MAGs. Briefly, we selected reference protein sequences from GenBank (nr database) using BLASTp and aligned them to the corresponding sequences predicted from the vOTUs with MAFFT (Katoh and Standley, 2013). The alignment was used as input for phylogenetic analyses with PHYML (Guindon and Gascuel, 2003) implemented in Geneious Prime 2022.1.1 (Biomatters Ltd.) using the LG evolutionary model and 500 bootstrap replicates. Whereas vOTUs 2, 61 and 62 seem to be related to tailed viruses isolated from Gram-negative bacteria, vOTUs 5 and 65 cluster with virus sequences obtained from Gram-positive bacteria belonging to the order Lactobacillales (Figure 3). Viral OTU2, which appeared both in the DNA and RNA metagenomes, clusters with phages from *Bartonella*, a facultative symbiont of the honey bee, and vOTUs 61 and 62 are related to a *Gilliamella* phage isolated from bumble bees in China. Proteobacteria such as *Bartonella* and *Gilliamella* are not as abundant in the *M. quadrifasciata* microbiome as yet unidentified Acetobacteraceae, in contrast to the Lactobacillales, that are highly abundant and diverse (Díaz *et al*., 2017; Cerqueira *et al*., 2021; Haag *et al*., 2023; Sarton-Lohéac *et al*., 2023).

**Figure 3 -:**
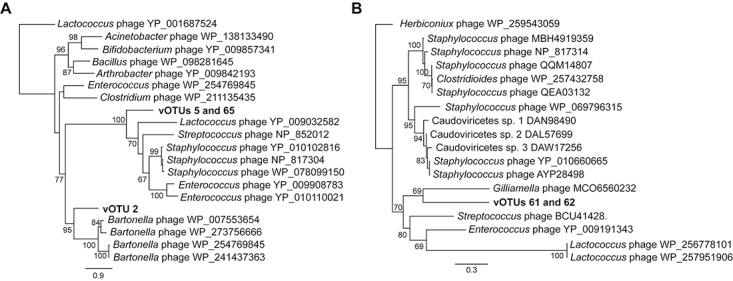
Maximum Likelihood phylogenies obtained using DNA encapsidation (A) and major capsid (B) proteins (see text for details). Proteins predicted from vOTUs 5 and 65, as well as vOTUs 61 and 62 were identical, and therefore correspond to a single branch on their respective phylogenetic trees.

Surprisingly, none of the sequences from our phageomes could be classified as the same phages previously characterized in honey bees (Bonilla-Rosso *et al*., 2020; Deboutte *et al*., 2020; Busby *et al*., 2022). Nonetheless, Caudoviricetes were also the most abundant phages identified in the honey bee studies, which were not designed to assess matters related to bee health. Our study compares the phageomes of stingless bees from the same species sampled simultaneously from the same location, but differing in their health status. We only detected Caudoviricetes phages in diseased bees, leading us to reason that these phages might be implicated in their health. One hypothesis is that the virulent tailed phages opportunistically become more abundant in diseased *M. quadrifasciata* due to an overall increase in the amount of bacteria in the gut. Indeed, in our previous study we found that bacterial counts estimated by 16S qPCR increased between January and March, but we did not find significant differences between diseased and healthy bees (Haag *et al*., 2023). An alternative but not mutually exclusive hypothesis is that, due to host specificity, tailed viruses disrupt the equilibrium of the bacterial community, and that microbiome dysbiosis negatively affects host homeostasis. We did find in previous studies of the *M. quadrifasciata* microbiome based on metabarcoding that the Lactobacillales reached their lowest relative abundance during the outbreak period, but again there were no significant differences between diseased and healthy bees (Haag *et al*., 2023).

It is possible that the dynamics of the microbiome, including bacteriophages, have a longer-term effect on bee health that we did not bring to light by looking at a single outbreak. Indeed, by studying two successive outbreaks (2014 and 2015), we showed a significant interaction effect of sampling year and health status on microbiome composition (Díaz *et al*., 2017), implying that the microbiome profile of diseased bees is not the same for every outbreak. To obtain a more realistic view about the role of bacteriophages in the *M. quadrifasciata* microbiome, and its relationship with the annual syndrome, we need to work with deeper sequencing efforts. It would be possible to use a metagenomic strategy to characterize the phageomes and bacteriomes simultaneously in successive years. Nevertheless, to our knowledge, this is the first report of bacteriophages from a stingless bee microbiome. We expect that, in the near future, with proper sampling and deep sequencing, we will be able to clarify how phages control bacterial densities in the *M. quadrifasciata* gut and ultimately influence their health. 

## Supplementary material

The following online material is available for this article:

Table S1 - Summary of DNA and RNA contig taxonomy.

Table S2 - Metagenome coverage and contig length.
