# Supplementary material for: Tailed bacteriophages (Caudoviricetes) dominate the microbiome of a diseased stingless bee
Source: Genet Mol Biol. 2024 Jan 19;46(3 Suppl 1):e20230120. doi: 10.1590/1678-4685-GMB-2023-0120 (PMC10802228; doi:10.1590/1678-4685-GMB-2023-0120)
Supplement: Table S1 - [file 1415-4757-GMB-46-03-s1-e20230120-s1.pdf]

Genetics and Molecular Biology

https://doi.org/10.1590/1678-4685-GMB-2023-0120

Supplementary Material to “Tailed bacteriophages (Caudoviricetes) dominate the microbiome of a diseased stingless bee”

Table S1 – Summary of DNA and RNA contig taxonomy.

geNomad

MAG	length	n_genes	n_hallmarks	taxonomy
58206_11_total_counts:_43766_Seed:_5_K:_25_length:_3937	3937	5	1	Viruses;Duplodnaviria;Heunggongvirae;Uroviricota;Caudoviricetes
87883_6_total_counts:_7909_Seed:_7_K:_25_length:_1336	1336	3	1	Viruses;Duplodnaviria;Heunggongvirae;Uroviricota;Caudoviricetes
29416_19_total_counts:_128119_Seed:_2_K:_25_length:_6724	6724	9	0	Viruses;Duplodnaviria;Heunggongvirae;Uroviricota;Caudoviricetes
145548_14_total_counts:_225461_Seed:_2_K:_25_length:_16338	16338	15	1	Viruses;Duplodnaviria;Heunggongvirae;Uroviricota;Caudoviricetes
145736_12_total_counts:_14774_Seed:_6_K:_25_length:_1211	1211	2	1	Viruses;Duplodnaviria;Heunggongvirae;Uroviricota;Caudoviricetes
146790_4_total_counts:_3112_Seed:_6_K:_25_length:_754	754	1	1	Viruses;Duplodnaviria;Heunggongvirae;Uroviricota;Caudoviricetes
145684_4_total_counts:_4230_Seed:_2_K:_25_length:_984	984	2	1	Viruses;Duplodnaviria;Heunggongvirae;Uroviricota;Caudoviricetes
58070_3_total_counts:_2596_Seed:_4_K:_25_length:_848	848	2	1	Viruses;Duplodnaviria;Heunggongvirae;Uroviricota;Caudoviricetes
150749_4_total_counts:_1179_Seed:_6_K:_25_length:_342	342	1	1	Viruses;Duplodnaviria;Heunggongvirae;Uroviricota;Caudoviricetes
59363_5_total_counts:_3352_Seed:_10_K:_25_length:_636	636	2	2	Viruses;Duplodnaviria;Heunggongvirae;Uroviricota;Caudoviricetes
146551_4_total_counts:_4589_Seed:_7_K:_25_length:_1126	1126	4	1	Viruses;Duplodnaviria;Heunggongvirae;Uroviricota;Caudoviricetes
29426_4_total_counts:_3393_Seed:_5_K:_25_length:_879	879	1	1	Viruses;Duplodnaviria;Heunggongvirae;Uroviricota;Caudoviricetes
93068_2_total_counts:_738_Seed:_3_K:_25_length:_357	357	1	1	Viruses;Duplodnaviria;Heunggongvirae;Uroviricota;Caudoviricetes
116451_5_total_counts:_4758_Seed:_5_K:_25_length:_1031	1031	2	1	Viruses;Duplodnaviria;Heunggongvirae;Uroviricota;Caudoviricetes
58735_5_total_counts:_3285_Seed:_7_K:_25_length:_679	679	3	1	Viruses;Duplodnaviria;Heunggongvirae;Uroviricota;Caudoviricetes
30547_13_total_counts:_6700_Seed:_21_K:_25_length:_552	552	1	1	Viruses;Duplodnaviria;Heunggongvirae;Uroviricota;Caudoviricetes
88020_5_total_counts:_4331_Seed:_7_K:_25_length:_985	985	2	1	Viruses;Duplodnaviria;Heunggongvirae;Uroviricota;Caudoviricetes
58062_2819_total_counts:_10018172_Seed:_3_K:_25_length:_3578	3578	6	0	Viruses;Duplodnaviria;Heunggongvirae;Uroviricota;Caudoviricetes;Rountreeviridae
116252_11_total_counts:_45454_Seed:_6_K:_25_length:_4300	4300	5	0	Viruses;Duplodnaviria;Heunggongvirae;Uroviricota;Caudoviricetes

MAG	length	n_genes	n_hallmarks	taxonomy
906_7_total_counts:_14133_Seed:_6_K:_25_length:_1980	1980	2	1	Viruses;Duplodnaviria;Heunggongvirae;Uroviricota;Caudoviricetes
30645_5_total_counts:_2820_Seed:_4_K:_25_length:_589	589	2	1	Viruses;Duplodnaviria;Heunggongvirae;Uroviricota;Caudoviricetes
147384_7_total_counts:_6615_Seed:_6_K:_25_length:_1012	1012	2	1	Viruses;Duplodnaviria;Heunggongvirae;Uroviricota;Caudoviricetes
87966_7_total_counts:_3026_Seed:_6_K:_25_length:_480	480	1	1	Viruses;Duplodnaviria;Heunggongvirae;Uroviricota;Caudoviricetes
150732_2_total_counts:_730_Seed:_4_K:_25_length:_327	327	1	1	Viruses;Duplodnaviria;Heunggongvirae;Uroviricota;Caudoviricetes
119947_3_total_counts:_890_Seed:_6_K:_25_length:_325	325	1	1	Viruses;Duplodnaviria;Heunggongvirae;Uroviricota;Caudoviricetes
4581_4_total_counts:_1133_Seed:_5_K:_25_length:_322	322	1	1	Viruses;Duplodnaviria;Heunggongvirae;Uroviricota;Caudoviricetes
146107_7_total_counts:_6223_Seed:_2_K:_25_length:_980	980	2	1	Viruses;Duplodnaviria;Heunggongvirae;Uroviricota;Caudoviricetes
87678_6_total_counts:_5258_Seed:_9_K:_25_length:_941	941	2	1	Viruses;Duplodnaviria;Heunggongvirae;Uroviricota;Caudoviricetes
115802_49_total_counts:_671701_Seed:_2_K:_25_length:_13606	13606	16	1	Viruses;Duplodnaviria;Heunggongvirae;Uroviricota;Caudoviricetes
1737_4_total_counts:_1522_Seed:_2_K:_25_length:_431	431	1	1	Viruses;Duplodnaviria;Heunggongvirae;Uroviricota;Caudoviricetes
146821_5_total_counts:_2458_Seed:_2_K:_25_length:_524	524	1	1	Viruses;Duplodnaviria;Heunggongvirae;Uroviricota;Caudoviricetes
2_3069_total_counts:_35374508_Seed:_6_K:_25_length:_11549	11549	13	0	Viruses;Duplodnaviria;Heunggongvirae;Uroviricota;Caudoviricetes;Rountreeviridae
118456_5_total_counts:_4582_Seed:_2_K:_25_length:_965	965	2	1	Viruses;Duplodnaviria;Heunggongvirae;Uroviricota;Caudoviricetes
29135_15_total_counts:_37696_Seed:_18_K:_25_length:_2471	2471	3	2	Viruses;Duplodnaviria;Heunggongvirae;Uroviricota;Caudoviricetes
147822_7_total_counts:_2845_Seed:_4_K:_25_length:_412	412	1	1	Viruses;Duplodnaviria;Heunggongvirae;Uroviricota;Caudoviricetes
34695_2_total_counts:_691_Seed:_2_K:_25_length:_327	327	1	1	Viruses;Duplodnaviria;Heunggongvirae;Uroviricota;Caudoviricetes
155772_2_total_counts:_737_Seed:_2_K:_25_length:_368	368	1	1	Viruses;Duplodnaviria;Heunggongvirae;Uroviricota;Caudoviricetes
146862_3_total_counts:_1436_Seed:_3_K:_25_length:_465	465	1	1	Viruses;Duplodnaviria;Heunggongvirae;Uroviricota;Caudoviricetes
58124_1283_total_counts:_2159833_Seed:_5_K:_25_length:_1707	1707	2	1	Viruses;Duplodnaviria;Heunggongvirae;Uroviricota;Caudoviricetes
152065_4_total_counts:_2093_Seed:_6_K:_25_length:_561	561	1	1	Viruses;Duplodnaviria;Heunggongvirae;Uroviricota;Caudoviricetes
62821_2_total_counts:_663_Seed:_2_K:_25_length:_372	372	1	1	Viruses;Duplodnaviria;Heunggongvirae;Uroviricota;Caudoviricetes
146197_4_total_counts:_3045_Seed:_2_K:_25_length:_808	808	3	1	Viruses;Duplodnaviria;Heunggongvirae;Uroviricota;Caudoviricetes
118886_3_total_counts:_1884_Seed:_4_K:_25_length:_577	577	1	1	Viruses;Duplodnaviria;Heunggongvirae;Uroviricota;Caudoviricetes
34480_2_total_counts:_589_Seed:_2_K:_25_length:_356	356	1	1	Viruses;Duplodnaviria;Heunggongvirae;Uroviricota;Caudoviricetes
119828_3_total_counts:_1364_Seed:_5_K:_25_length:_451	451	1	1	Viruses;Duplodnaviria;Heunggongvirae;Uroviricota;Caudoviricetes
92269_2_total_counts:_813_Seed:_3_K:_25_length:_450	450	1	1	Viruses;Duplodnaviria;Heunggongvirae;Uroviricota;Caudoviricetes
33002_2_total_counts:_1040_Seed:_3_K:_25_length:_450	450	1	1	Viruses;Duplodnaviria;Heunggongvirae;Uroviricota;Caudoviricetes
29119_2756_total_counts:_7460053_Seed:_2_K:_25_length:_2731	2731	5	0	Viruses;Duplodnaviria;Heunggongvirae;Uroviricota;Caudoviricetes
160_2_total_counts:_1030_Seed:_3_K:_25_length:_495	495	1	1	Viruses;Duplodnaviria;Heunggongvirae;Uroviricota;Caudoviricetes
147066_5_total_counts:_1636_Seed:_3_K:_25_length:_324	324	1	1	Viruses;Duplodnaviria;Heunggongvirae;Uroviricota;Caudoviricetes
146574_4_total_counts:_2199_Seed:_2_K:_25_length:_626	626	1	1	Viruses;Duplodnaviria;Heunggongvirae;Uroviricota;Caudoviricetes
119050_10_total_counts:_6075_Seed:_15_K:_25_length:_629	629	1	1	Viruses;Duplodnaviria;Heunggongvirae;Uroviricota;Caudoviricetes
63434_2_total_counts:_490_Seed:_2_K:_25_length:_331	331	1	1	Viruses;Duplodnaviria;Heunggongvirae;Uroviricota;Caudoviricetes
NODE_3_length_5385_cov_8.33527	5385	14	0	Viruses;Duplodnaviria;Heunggongvirae;Uroviricota;Caudoviricetes
NODE_2_length_5413_cov_6.22714	5413	10	0	Viruses;Duplodnaviria;Heunggongvirae;Uroviricota;Caudoviricetes

MAG	length	n_genes	n_hallmarks	taxonomy
NODE_12_length_2790_cov_15.8786	2790	8	0	Viruses;Duplodnaviria;Heunggongvirae;Uroviricota;Caudoviricetes
NODE_19_length_2673_cov_11.95	2673	7	0	Viruses;Duplodnaviria;Heunggongvirae;Uroviricota;Caudoviricetes
NODE_7_length_3822_cov_11.8471	3822	11	1	Viruses;Duplodnaviria;Heunggongvirae;Uroviricota;Caudoviricetes
NODE_358_length_449_cov_1.63959	449	1	1	Viruses;Duplodnaviria;Heunggongvirae;Uroviricota;Caudoviricetes
NODE_14_length_2710_cov_11.145	2710	2	0	Viruses;Duplodnaviria;Heunggongvirae;Uroviricota;Caudoviricetes
NODE_91_length_1174_cov_9.41555	1174	2	1	Viruses;Duplodnaviria;Heunggongvirae;Uroviricota;Caudoviricetes
NODE_235_length_579_cov_1.58779	579	1	1	Viruses;Duplodnaviria;Heunggongvirae;Uroviricota;Caudoviricetes
NODE_306_length_511_cov_3.29825	511	1	1	Viruses;Duplodnaviria;Heunggongvirae;Uroviricota;Caudoviricetes
NODE_241_length_567_cov_5.27539	567	1	1	Viruses;Duplodnaviria;Heunggongvirae;Uroviricota;Caudoviricetes
NODE_16_length_2705_cov_15.2894	2705	6	0	Viruses;Duplodnaviria;Heunggongvirae;Uroviricota;Caudoviricetes
NODE_1_length_14405_cov_25.8403	14405	19	1	Viruses;Duplodnaviria;Heunggongvirae;Uroviricota;Caudoviricetes
NODE_9_length_2927_cov_9.01358	2927	9	0	Viruses;Duplodnaviria;Heunggongvirae;Uroviricota;Caudoviricetes
NODE_31_length_2251_cov_3.51685	2251	6	1	Viruses;Duplodnaviria;Heunggongvirae;Uroviricota;Caudoviricetes
NODE_18_length_2681_cov_5.18012	2681	3	0	Viruses;Duplodnaviria;Heunggongvirae;Uroviricota;Caudoviricetes
NODE_245_length_564_cov_2.4165	564	2	1	Viruses;Duplodnaviria;Heunggongvirae;Uroviricota;Caudoviricetes
NODE_97_length_1108_cov_2.96771	1108	2	1	Viruses;Duplodnaviria;Heunggongvirae;Uroviricota;Caudoviricetes
NODE_164_length_751_cov_2.38075	751	2	1	Viruses;Duplodnaviria;Heunggongvirae;Uroviricota;Caudoviricetes
NODE_86_length_1202_cov_8.1578	1202	2	1	Viruses;Duplodnaviria;Heunggongvirae;Uroviricota;Caudoviricetes
NODE_22_length_2568_cov_7.01074	2568	9	0	Viruses;Duplodnaviria;Heunggongvirae;Uroviricota;Caudoviricetes
NODE_229_length_586_cov_3.24482	586	2	1	Viruses;Duplodnaviria;Heunggongvirae;Uroviricota;Caudoviricetes
NODE_371_length_434_cov_2.53298	434	1	1	Viruses;Duplodnaviria;Heunggongvirae;Uroviricota;Caudoviricetes
NODE_670_length_331_cov_1.9058	331	2	1	Viruses;Duplodnaviria;Heunggongvirae;Uroviricota;Caudoviricetes
NODE_620_length_342_cov_2.31359	342	1	1	Viruses;Duplodnaviria;Heunggongvirae;Uroviricota;Caudoviricetes
NODE_38_length_2005_cov_1102.3	2005	2	1	Viruses;Duplodnaviria;Heunggongvirae;Uroviricota;Caudoviricetes
NODE_204_length_622_cov_2.08289	622	1	1	Viruses;Duplodnaviria;Heunggongvirae;Uroviricota;Caudoviricetes
NODE_8_length_3348_cov_7.53325	3348	9	0	Viruses;Duplodnaviria;Heunggongvirae;Uroviricota;Caudoviricetes
NODE_488_length_376_cov_4.82866	376	1	1	Viruses;Duplodnaviria;Heunggongvirae;Uroviricota;Caudoviricetes
NODE_59_length_1601_cov_1305.56	1601	3	1	Viruses;Duplodnaviria;Heunggongvirae;Uroviricota;Caudoviricetes

virSorter2

MAG	max_score_group	length	hallmark
244_4_total_counts:_3220_Seed:_9_K:_25_length:_772||full	dsDNAphage	771	1

MAG	max_score_group	length	hallmark
145548_14_total_counts:_225461_Seed:_2_K:_25_length:_16338||full	dsDNAphage	16336	0
58124_1283_total_counts:_2159833_Seed:_5_K:_25_length:_1707||full	dsDNAphage	1392	1
146197_4_total_counts:_3045_Seed:_2_K:_25_length:_808||full	dsDNAphage	806	2
29135_15_total_counts:_37696_Seed:_18_K:_25_length:_2471||full	dsDNAphage	1973	1
115802_49_total_counts:_671701_Seed:_2_K:_25_length:_13606||full	dsDNAphage	13455	4
87573_5_total_counts:_8642_Seed:_3_K:_25_length:_1816||full	dsDNAphage	1813	1
145684_4_total_counts:_4230_Seed:_2_K:_25_length:_984||full	dsDNAphage	947	2
58062_2819_total_counts:_10018172_Seed:_3_K:_25_length:_3578||full	dsDNAphage	3576	3
58206_11_total_counts:_43766_Seed:_5_K:_25_length:_3937||full	dsDNAphage	3732	1
2_3069_total_counts:_35374508_Seed:_6_K:_25_length:_11549||full	dsDNAphage	11063	3
65_6_total_counts:_4013_Seed:_2_K:_25_length:_656||lt2gene	dsDNAphage	656	1
160_2_total_counts:_1030_Seed:_3_K:_25_length:_495||lt2gene	dsDNAphage	495	1
489_8_total_counts:_6985_Seed:_15_K:_25_length:_899||lt2gene	dsDNAphage	899	1
839_6_total_counts:_1702_Seed:_2_K:_25_length:_318||lt2gene	dsDNAphage	318	1
1589_3_total_counts:_1127_Seed:_6_K:_25_length:_436||lt2gene	dsDNAphage	436	1
1737_4_total_counts:_1522_Seed:_2_K:_25_length:_431||lt2gene	dsDNAphage	431	1
1808_3_total_counts:_2394_Seed:_2_K:_25_length:_818||lt2gene	dsDNAphage	818	1
2791_4_total_counts:_992_Seed:_5_K:_25_length:_304||lt2gene	dsDNAphage	304	1
3104_4_total_counts:_2592_Seed:_3_K:_25_length:_673||lt2gene	dsDNAphage	673	1
4581_4_total_counts:_1133_Seed:_5_K:_25_length:_322||lt2gene	dsDNAphage	322	1
4907_4_total_counts:_1091_Seed:_5_K:_25_length:_303||lt2gene	dsDNAphage	303	1
5118_5_total_counts:_3299_Seed:_3_K:_25_length:_722||lt2gene	dsDNAphage	722	1
5135_3_total_counts:_781_Seed:_6_K:_25_length:_311||lt2gene	dsDNAphage	311	1
9077_3_total_counts:_798_Seed:_3_K:_25_length:_312||lt2gene	dsDNAphage	312	1
29426_4_total_counts:_3393_Seed:_5_K:_25_length:_879||lt2gene	dsDNAphage	879	1
29514_4_total_counts:_2397_Seed:_2_K:_25_length:_651||lt2gene	dsDNAphage	651	1
29989_4_total_counts:_1646_Seed:_3_K:_25_length:_392||lt2gene	dsDNAphage	392	1
30242_10_total_counts:_7809_Seed:_16_K:_25_length:_836||lt2gene	dsDNAphage	836	1
30547_13_total_counts:_6700_Seed:_21_K:_25_length:_552||lt2gene	dsDNAphage	552	1
31238_3_total_counts:_1025_Seed:_2_K:_25_length:_346||lt2gene	dsDNAphage	346	1
31951_4_total_counts:_1233_Seed:_2_K:_25_length:_360||lt2gene	dsDNAphage	360	1
33002_2_total_counts:_1040_Seed:_3_K:_25_length:_450||lt2gene	dsDNAphage	450	1
33456_2_total_counts:_799_Seed:_4_K:_25_length:_376||lt2gene	dsDNAphage	376	1
34695_2_total_counts:_691_Seed:_2_K:_25_length:_327||lt2gene	dsDNAphage	327	1

MAG	max_score_group	length	hallmark
35477_3_total_counts:_1262_Seed:_4_K:_25_length:_401||lt2gene	dsDNAphage	401	1
35784_3_total_counts:_1182_Seed:_2_K:_25_length:_403||lt2gene	dsDNAphage	403	1
58070_3_total_counts:_2596_Seed:_4_K:_25_length:_848||lt2gene	dsDNAphage	848	1
58655_3_total_counts:_818_Seed:_4_K:_25_length:_302||lt2gene	dsDNAphage	302	1
58672_3_total_counts:_2088_Seed:_3_K:_25_length:_747||lt2gene	dsDNAphage	747	1
59767_7_total_counts:_4925_Seed:_7_K:_25_length:_701||lt2gene	dsDNAphage	701	1
60296_2_total_counts:_794_Seed:_2_K:_25_length:_368||lt2gene	dsDNAphage	368	1
61764_3_total_counts:_1169_Seed:_2_K:_25_length:_378||lt2gene	dsDNAphage	378	1
62462_5_total_counts:_1641_Seed:_3_K:_25_length:_339||lt2gene	dsDNAphage	339	1
62821_2_total_counts:_663_Seed:_2_K:_25_length:_372||lt2gene	dsDNAphage	372	1
63434_2_total_counts:_490_Seed:_2_K:_25_length:_331||lt2gene	dsDNAphage	331	1
64062_2_total_counts:_761_Seed:_2_K:_25_length:_345||lt2gene	dsDNAphage	345	1
64890_2_total_counts:_878_Seed:_2_K:_25_length:_407||lt2gene	dsDNAphage	407	1
87683_5_total_counts:_2612_Seed:_4_K:_25_length:_604||lt2gene	dsDNAphage	604	1
87688_7_total_counts:_2137_Seed:_3_K:_25_length:_333||lt2gene	dsDNAphage	333	1
87902_4_total_counts:_1124_Seed:_3_K:_25_length:_327||lt2gene	dsDNAphage	327	1
87966_7_total_counts:_3026_Seed:_6_K:_25_length:_480||lt2gene	dsDNAphage	480	1
88020_5_total_counts:_4331_Seed:_7_K:_25_length:_985||lt2gene	dsDNAphage	985	1
88138_3_total_counts:_1332_Seed:_2_K:_25_length:_498||lt2gene	dsDNAphage	498	1
88229_6_total_counts:_5692_Seed:_2_K:_25_length:_1024||lt2gene	dsDNAphage	1024	1
88535_5_total_counts:_3188_Seed:_2_K:_25_length:_709||lt2gene	dsDNAphage	709	1
89109_5_total_counts:_2537_Seed:_4_K:_25_length:_541||lt2gene	dsDNAphage	541	1
90635_3_total_counts:_848_Seed:_5_K:_25_length:_312||lt2gene	dsDNAphage	312	1
91422_5_total_counts:_7244_Seed:_6_K:_25_length:_1453||lt2gene	dsDNAphage	1453	1
92269_2_total_counts:_813_Seed:_3_K:_25_length:_450||lt2gene	dsDNAphage	450	1
93068_2_total_counts:_738_Seed:_3_K:_25_length:_357||lt2gene	dsDNAphage	357	1
95022_2_total_counts:_512_Seed:_2_K:_25_length:_312||lt2gene	dsDNAphage	312	1
116247_6_total_counts:_3553_Seed:_2_K:_25_length:_661||lt2gene	dsDNAphage	661	1
117147_6_total_counts:_3442_Seed:_3_K:_25_length:_595||lt2gene	dsDNAphage	595	1
118198_5_total_counts:_1677_Seed:_2_K:_25_length:_369||lt2gene	dsDNAphage	369	1
118886_3_total_counts:_1884_Seed:_4_K:_25_length:_577||lt2gene	dsDNAphage	577	1
119038_2_total_counts:_534_Seed:_2_K:_25_length:_349||lt2gene	dsDNAphage	349	1
119050_10_total_counts:_6075_Seed:_15_K:_25_length:_629||lt2gene	dsDNAphage	629	1
119564_3_total_counts:_930_Seed:_3_K:_25_length:_345||lt2gene	dsDNAphage	345	1
120792_3_total_counts:_859_Seed:_3_K:_25_length:_338||lt2gene	dsDNAphage	338	1
145736_12_total_counts:_14774_Seed:_6_K:_25_length:_1211||lt2gen	dsDNAphage	1211	2

MAG<br>e	max_score_group	length	hallmark
146281_6_total_counts:_5714_Seed:_15_K:_25_length:_1054||lt2gene	dsDNAphage	1054	1
146417_4_total_counts:_1476_Seed:_2_K:_25_length:_408||lt2gene	dsDNAphage	408	1
146509_3_total_counts:_878_Seed:_2_K:_25_length:_312||lt2gene	dsDNAphage	312	1
146541_4_total_counts:_1219_Seed:_2_K:_25_length:_343||lt2gene	dsDNAphage	343	1
146574_4_total_counts:_2199_Seed:_2_K:_25_length:_626||lt2gene	dsDNAphage	626	1
146623_4_total_counts:_2347_Seed:_5_K:_25_length:_639||lt2gene	dsDNAphage	639	1
146706_9_total_counts:_7470_Seed:_3_K:_25_length:_826||lt2gene	dsDNAphage	826	1
146790_4_total_counts:_3112_Seed:_6_K:_25_length:_754||lt2gene	dsDNAphage	754	1
146821_5_total_counts:_2458_Seed:_2_K:_25_length:_524||lt2gene	dsDNAphage	524	1
146862_3_total_counts:_1436_Seed:_3_K:_25_length:_465||lt2gene	dsDNAphage	465	1
147066_5_total_counts:_1636_Seed:_3_K:_25_length:_324||lt2gene	dsDNAphage	324	1
147164_6_total_counts:_5790_Seed:_8_K:_25_length:_1028||lt2gene	dsDNAphage	1028	1
147249_4_total_counts:_1593_Seed:_6_K:_25_length:_456||lt2gene	dsDNAphage	456	1
147319_5_total_counts:_4372_Seed:_2_K:_25_length:_958||lt2gene	dsDNAphage	958	1
147376_5_total_counts:_1753_Seed:_7_K:_25_length:_376||lt2gene	dsDNAphage	376	1
147384_7_total_counts:_6615_Seed:_6_K:_25_length:_1012||lt2gene	dsDNAphage	1012	1
147822_7_total_counts:_2845_Seed:_4_K:_25_length:_412||lt2gene	dsDNAphage	412	1
148545_5_total_counts:_4407_Seed:_4_K:_25_length:_906||lt2gene	dsDNAphage	906	1
149082_2_total_counts:_673_Seed:_5_K:_25_length:_316||lt2gene	dsDNAphage	316	1
149322_3_total_counts:_1310_Seed:_4_K:_25_length:_469||lt2gene	dsDNAphage	469	1
150732_2_total_counts:_730_Seed:_4_K:_25_length:_327||lt2gene	dsDNAphage	327	1
151973_2_total_counts:_631_Seed:_5_K:_25_length:_306||lt2gene	dsDNAphage	306	1
152065_4_total_counts:_2093_Seed:_6_K:_25_length:_561||lt2gene	dsDNAphage	561	1
152075_4_total_counts:_1531_Seed:_5_K:_25_length:_416||lt2gene	dsDNAphage	416	1
155087_2_total_counts:_779_Seed:_2_K:_25_length:_423||lt2gene	dsDNAphage	423	1
155141_2_total_counts:_477_Seed:_2_K:_25_length:_306||lt2gene	dsDNAphage	306	1
155663_2_total_counts:_740_Seed:_2_K:_25_length:_417||lt2gene	dsDNAphage	417	1
155772_2_total_counts:_737_Seed:_2_K:_25_length:_368||lt2gene	dsDNAphage	368	1
155933_2_total_counts:_633_Seed:_4_K:_25_length:_307||lt2gene	dsDNAphage	307	1
156327_4_total_counts:_1421_Seed:_2_K:_25_length:_394||lt2gene	dsDNAphage	394	1
161001_2_total_counts:_637_Seed:_2_K:_25_length:_374||lt2gene	dsDNAphage	374	1
33351_4_total_counts:_1147_Seed:_8_K:_25_length:_342||lt2gene	dsDNAphage	342	1
59363_5_total_counts:_3352_Seed:_10_K:_25_length:_636||lt2gene	dsDNAphage	636	1
59400_5_total_counts:_3435_Seed:_4_K:_25_length:_714||lt2gene	dsDNAphage	714	1
62167_2_total_counts:_983_Seed:_3_K:_25_length:_465||lt2gene	dsDNAphage	465	1

MAG	max_score_group	length	hallmark
62682_5_total_counts:_1539_Seed:_2_K:_25_length:_321||lt2gene	dsDNAphage	321	1
87678_6_total_counts:_5258_Seed:_9_K:_25_length:_941||lt2gene	dsDNAphage	941	1
116472_2_total_counts:_909_Seed:_2_K:_25_length:_582||lt2gene	dsDNAphage	582	1
145591_3_total_counts:_3090_Seed:_2_K:_25_length:_1074||lt2gene	dsDNAphage	1074	1
146684_12_total_counts:_7542_Seed:_3_K:_25_length:_676||lt2gene	dsDNAphage	676	1
149274_2_total_counts:_1187_Seed:_3_K:_25_length:_642||lt2gene	dsDNAphage	642	1
154270_3_total_counts:_793_Seed:_4_K:_25_length:_320||lt2gene	dsDNAphage	320	1
162105_2_total_counts:_849_Seed:_2_K:_25_length:_395||lt2gene	dsDNAphage	395	1
NODE_1_length_14405_cov_25.8403||full	dsDNAphage	14403	4
NODE_2_length_5413_cov_6.22714||full	dsDNAphage	5411	0
NODE_3_length_5385_cov_8.33527||full	dsDNAphage	5372	0
NODE_7_length_3822_cov_11.8471||full	dsDNAphage	3820	0
NODE_8_length_3348_cov_7.53325||full	dsDNAphage	3346	0
NODE_19_length_2673_cov_11.95||full	dsDNAphage	2671	1
NODE_26_length_2424_cov_5.68763||full	dsDNAphage	2422	1
NODE_28_length_2286_cov_6.76109||full	dsDNAphage	2284	1
NODE_40_length_1902_cov_3.04169||full	dsDNAphage	1899	3
NODE_54_length_1661_cov_6.05168||full	dsDNAphage	1659	1
NODE_55_length_1650_cov_4.20313||full	dsDNAphage	1647	1
NODE_86_length_1202_cov_8.1578||full	dsDNAphage	1176	1
NODE_177_length_702_cov_4.84853||full	dsDNAphage	701	1
NODE_14_length_2710_cov_11.145||lt2gene	dsDNAphage	2710	1
NODE_38_length_2005_cov_1102.3||lt2gene	dsDNAphage	2005	1
NODE_47_length_1772_cov_19.8427||lt2gene	dsDNAphage	1772	1
NODE_50_length_1707_cov_4.08414||lt2gene	dsDNAphage	1707	1
NODE_91_length_1174_cov_9.41555||lt2gene	dsDNAphage	1174	1
NODE_97_length_1108_cov_2.96771||lt2gene	dsDNAphage	1108	1
NODE_130_length_903_cov_4.64741||lt2gene	dsDNAphage	903	1
NODE_142_length_837_cov_2004.61||lt2gene	dsDNAphage	837	1
NODE_164_length_751_cov_2.38075||lt2gene	dsDNAphage	751	1
NODE_192_length_651_cov_2.01174||lt2gene	dsDNAphage	651	1
NODE_204_length_622_cov_2.08289||lt2gene	dsDNAphage	622	1
NODE_208_length_612_cov_13.7487||lt2gene	dsDNAphage	612	1
NODE_235_length_579_cov_1.58779||lt2gene	dsDNAphage	579	1
NODE_241_length_567_cov_5.27539||lt2gene	dsDNAphage	567	1
NODE_245_length_564_cov_2.4165||lt2gene	dsDNAphage	564	1

MAG	max_score_group	length	hallmark
NODE_249_length_562_cov_2.46351||lt2gene	dsDNAphage	562	1
NODE_257_length_556_cov_1608.69||lt2gene	dsDNAphage	556	1
NODE_261_length_553_cov_2.07229||lt2gene	dsDNAphage	553	1
NODE_262_length_553_cov_1.93173||lt2gene	dsDNAphage	553	1
NODE_291_length_525_cov_3.8||lt2gene	dsDNAphage	525	1
NODE_306_length_511_cov_3.29825||lt2gene	dsDNAphage	511	1
NODE_307_length_511_cov_1.53289||lt2gene	dsDNAphage	511	1
NODE_313_length_501_cov_2.73543||lt2gene	dsDNAphage	501	1
NODE_358_length_449_cov_1.63959||lt2gene	dsDNAphage	449	1
NODE_373_length_433_cov_3.62169||lt2gene	dsDNAphage	433	1
NODE_390_length_422_cov_1051.65||lt2gene	dsDNAphage	422	1
NODE_418_length_411_cov_1.70506||lt2gene	dsDNAphage	411	1
NODE_441_length_400_cov_1.93623||lt2gene	dsDNAphage	400	1
NODE_466_length_386_cov_1.8429||lt2gene	dsDNAphage	386	1
NODE_470_length_384_cov_4.27964||lt2gene	dsDNAphage	384	1
NODE_488_length_376_cov_4.82866||lt2gene	dsDNAphage	376	1
NODE_491_length_375_cov_3.20938||lt2gene	dsDNAphage	375	1
NODE_547_length_359_cov_2.16118||lt2gene	dsDNAphage	359	1
NODE_574_length_354_cov_1.08027||lt2gene	dsDNAphage	354	1
NODE_620_length_342_cov_2.31359||lt2gene	dsDNAphage	342	1
NODE_654_length_334_cov_2.48746||lt2gene	dsDNAphage	334	1
NODE_664_length_332_cov_2.11913||lt2gene	dsDNAphage	332	1
NODE_702_length_324_cov_1.64312||lt2gene	dsDNAphage	324	1
NODE_738_length_317_cov_1.98092||lt2gene	dsDNAphage	317	1
NODE_741_length_316_cov_2.0613||lt2gene	dsDNAphage	316	1
NODE_755_length_314_cov_1.40541||lt2gene	dsDNAphage	314	1
