# Supplementary material for: Tailed bacteriophages (Caudoviricetes) dominate the microbiome of a diseased stingless bee
Source: Genet Mol Biol. 2024 Jan 19;46(3 Suppl 1):e20230120. doi: 10.1590/1678-4685-GMB-2023-0120 (PMC10802228; doi:10.1590/1678-4685-GMB-2023-0120)
Supplement: Table S2 - [file 1415-4757-GMB-46-03-s1-e20230120-s2.pdf]

Genetics and Molecular Biology
https://doi.org/10.1590/1678-4685-GMB-2023-0120

Supplementary Material to "Tailed bacteriophages (Caudoviricetes) dominate the microbiome of a diseased stingless bee"

Table S2 – Metagenome coverage and contig length.

RNA metagenome

vOTU	coverage	contig/bin_id	length
vOTU_5	2704.15	2_3069_total_counts:_35374508_Seed:_6_K:_25_length:_11549	11549
vOTU_61	1521.98	58062_2819_total_counts:_10018172_Seed:_3_K:_25_length:_3578	3578
vOTU_62	1101.16	29119_2756_total_counts:_7460053_Seed:_2_K:_25_length:_2731	2731
vOTU_65	1079.46	58124_1283_total_counts:_2159833_Seed:_5_K:_25_length:_1707	1707
VOTU_2	51.8756	115802_49_total_counts:_671701_Seed:_2_K:_25_length:_13606	13606
VOTU_6	15.4469	29416_19_total_counts:_128119_Seed:_2_K:_25_length:_6724	6724
vOTU_68	13.8761	145736_12_total_counts:_14774_Seed:_6_K:_25_length:_1211	1211
VOTU_7	13.14205	Bin3	6408
vOTU_86	11.0275	30242_10_total_counts:_7809_Seed:_16_K:_25_length:_836	836
vOTU_4	10.8432	145548_14_total_counts:_225461_Seed:_2_K:_25_length:_16338	16338
vOTU_114	8.94746	30547_13_total_counts:_6700_Seed:_21_K:_25_length:_552	552
vOTU_60	8.75256	116252_11_total_counts:_45454_Seed:_6_K:_25_length:_4300	4300
vOTU_106	8.68203	119050_10_total_counts:_6075_Seed:_15_K:_25_length:_629	629
vOTU_98	7.4068	146684_12_total_counts:_7542_Seed:_3_K:_25_length:_676	676
vOTU_87	7.28087	146706_9_total_counts:_7470_Seed:_3_K:_25_length:_826	826
vOTU_96	6.1669	59767_7_total_counts:_4925_Seed:_7_K:_25_length:_701	701
vOTU_83	5.96552	489_8_total_counts:_6985_Seed:_15_K:_25_length:_899	899
vOTU_119	5.65833	87966_7_total_counts:_3026_Seed:_6_K:_25_length:_480	480
vOTU_63	5.37626	906_7_total_counts:_14133_Seed:_6_K:_25_length:_1980	1980
vOTU_132	5.32524	147822_7_total_counts:_2845_Seed:_4_K:_25_length:_412	412
vOTU_115	5.30869	89109_5_total_counts:_2537_Seed:_4_K:_25_length:_541	541
vOTU_109	5.2	117147_6_total_counts:_3442_Seed:_3_K:_25_length:_595	595
vOTU_81	5.03719	87678_6_total_counts:_5258_Seed:_9_K:_25_length:_941	941
vOTU_100	4.92133	116247_6_total_counts:_3553_Seed:_2_K:_25_length:_661	661
vOTU_168	4.85358	62682_5_total_counts:_1539_Seed:_2_K:_25_length:_321	321
vOTU_67	4.77994	87883_6_total_counts:_7909_Seed:_7_K:_25_length:_1336	1336
vOTU_74	4.76855	88229_6_total_counts:_5692_Seed:_2_K:_25_length:_1024	1024
vOTU_101	4.59756	65_6_total_counts:_4013_Seed:_2_K:_25_length:_656	656
vOTU_73	4.57685	147164_6_total_counts:_5790_Seed:_8_K:_25_length:_1028	1028
vOTU_105	4.56761	59363_5_total_counts:_3352_Seed:_10_K:_25_length:_636	636
vOTU_71	4.54649	146281_6_total_counts:_5714_Seed:_15_K:_25_length:_1054	1054
vOTU_166	4.51543	147066_5_total_counts:_1636_Seed:_3_K:_25_length:_324	324
vOTU_75	4.45949	147384_7_total_counts:_6615_Seed:_6_K:_25_length:_1012	1012

vOTU	coverage	contig/bin_id	length
vOTU_78	4.41122	146107_7_total_counts:_6223_Seed:_2_K:_25_length:_980	980
vOTU_141	4.3617	147376_5_total_counts:_1753_Seed:_7_K:_25_length:_376	376
vOTU_145	4.34417	118198_5_total_counts:_1677_Seed:_2_K:_25_length:_369	369
vOTU_66	4.31727	91422_5_total_counts:_7244_Seed:_6_K:_25_length:_1453	1453
vOTU_76	4.11878	88020_5_total_counts:_4331_Seed:_7_K:_25_length:_985	985
vOTU_160	4.10511	87688_7_total_counts:_2137_Seed:_3_K:_25_length:_333	333
vOTU_64	4.01652	87573_5_total_counts:_8642_Seed:_3_K:_25_length:_1816	1816
vOTU_94	3.92857	59400_5_total_counts:_3435_Seed:_4_K:_25_length:_714	714
vOTU_80	3.85282	147319_5_total_counts:_4372_Seed:_2_K:_25_length:_958	958
vOTU_156	3.72807	150749_4_total_counts:_1179_Seed:_6_K:_25_length:_342	342
vOTU_90	3.69301	244_4_total_counts:_3220_Seed:_9_K:_25_length:_772	772
vOTU_123	3.6886	147249_4_total_counts:_1593_Seed:_6_K:_25_length:_456	456
vOTU_116	3.57061	146821_5_total_counts:_2458_Seed:_2_K:_25_length:_524	524
vOTU_108	3.55298	87683_5_total_counts:_2612_Seed:_4_K:_25_length:_604	604
vOTU_79	3.49741	118456_5_total_counts:_4582_Seed:_2_K:_25_length:_965	965
vOTU_93	3.47645	5118_5_total_counts:_3299_Seed:_3_K:_25_length:_722	722
vOTU_133	3.46324	146417_4_total_counts:_1476_Seed:_2_K:_25_length:_408	408
vOTU_97	3.4271	58735_5_total_counts:_3285_Seed:_7_K:_25_length:_679	679
vOTU_139	3.42602	29989_4_total_counts:_1646_Seed:_3_K:_25_length:_392	392
vOTU_128	3.39443	1737_4_total_counts:_1522_Seed:_2_K:_25_length:_431	431
vOTU_77	3.28354	145684_4_total_counts:_4230_Seed:_2_K:_25_length:_984	984
vOTU_153	3.25507	119564_3_total_counts:_930_Seed:_3_K:_25_length:_345	345
vOTU_82	3.16556	148545_5_total_counts:_4407_Seed:_4_K:_25_length:_906	906
vOTU_72	3.13288	116451_5_total_counts:_4758_Seed:_5_K:_25_length:_1031	1031
vOTU_69	3.1119	146551_4_total_counts:_4589_Seed:_7_K:_25_length:_1126	1126
vOTU_104	3.0266	146623_4_total_counts:_2347_Seed:_5_K:_25_length:_639	639
vOTU_181	3.0033	4907_4_total_counts:_1091_Seed:_5_K:_25_length:_303	303
vOTU_157	3.00292	33351_4_total_counts:_1147_Seed:_8_K:_25_length:_342	342
vOTU_158	2.9587	62462_5_total_counts:_1641_Seed:_3_K:_25_length:_339	339
vOTU_121	2.91828	146862_3_total_counts:_1436_Seed:_3_K:_25_length:_465	465
vOTU_138	2.91371	156327_4_total_counts:_1421_Seed:_2_K:_25_length:_394	394
vOTU_112	2.88562	118886_3_total_counts:_1884_Seed:_4_K:_25_length:_577	577
vOTU_172	2.87821	146509_3_total_counts:_878_Seed:_2_K:_25_length:_312	312
vOTU_95	2.87165	88535_5_total_counts:_3188_Seed:_2_K:_25_length:_709	709
vOTU_170	2.85849	839_6_total_counts:_1702_Seed:_2_K:_25_length:_318	318
vOTU_120	2.85714	149322_3_total_counts:_1310_Seed:_4_K:_25_length:_469	469
vOTU_164	2.79511	87902_4_total_counts:_1124_Seed:_3_K:_25_length:_327	327
vOTU_135	2.79156	35784_3_total_counts:_1182_Seed:_2_K:_25_length:_403	403
vOTU_91	2.77321	146790_4_total_counts:_3112_Seed:_6_K:_25_length:_754	754
vOTU_107	2.72045	146574_4_total_counts:_2199_Seed:_2_K:_25_length:_626	626
vOTU_99	2.68796	3104_4_total_counts:_2592_Seed:_3_K:_25_length:_673	673
vOTU_89	2.6547	146197_4_total_counts:_3045_Seed:_2_K:_25_length:_808	808
vOTU_102	2.62826	29514_4_total_counts:_2397_Seed:_2_K:_25_length:_651	651
vOTU_117	2.60241	88138_3_total_counts:_1332_Seed:_2_K:_25_length:_498	498
vOTU_88	2.55623	1808_3_total_counts:_2394_Seed:_2_K:_25_length:_818	818
vOTU_165	2.52923	119947_3_total_counts:_890_Seed:_6_K:_25_length:_325	325
vOTU_180	2.51974	2791_4_total_counts:_992_Seed:_5_K:_25_length:_304	304
vOTU_127	2.49083	1589_3_total_counts:_1127_Seed:_6_K:_25_length:_436	436
vOTU_148	2.46667	31951_4_total_counts:_1233_Seed:_2_K:_25_length:_360	360
vOTU_131	2.43269	152075_4_total_counts:_1531_Seed:_5_K:_25_length:_416	416
vOTU_174	2.39103	9077_3_total_counts:_798_Seed:_3_K:_25_length:_312	312
vOTU_146	2.38315	155772_2_total_counts:_737_Seed:_2_K:_25_length:_368	368
vOTU_124	2.37694	119828_3_total_counts:_1364_Seed:_5_K:_25_length:_451	451
vOTU_84	2.37429	29426_4_total_counts:_3393_Seed:_5_K:_25_length:_879	879
vOTU_134	2.34889	64890_2_total_counts:_878_Seed:_2_K:_25_length:_407	407
vOTU_113	2.31016	152065_4_total_counts:_2093_Seed:_6_K:_25_length:_561	561
vOTU_92	2.29987	58672_3_total_counts:_2088_Seed:_3_K:_25_length:_747	747

vOTU	coverage	contig/bin_id	length
vOTU_155	2.27988	146541_4_total_counts:_1219_Seed:_2_K:_25_length:_343	343
vOTU_125	2.27556	33002_2_total_counts:_1040_Seed:_3_K:_25_length:_450	450
vOTU_70	2.25326	145591_3_total_counts:_3090_Seed:_2_K:_25_length:_1074	1074
vOTU_137	2.21013	162105_2_total_counts:_849_Seed:_2_K:_25_length:_395	395
vOTU_152	2.03757	31238_3_total_counts:_1025_Seed:_2_K:_25_length:_346	346
vOTU_173	2.01923	90635_3_total_counts:_848_Seed:_5_K:_25_length:_312	312
vOTU_149	1.96078	93068_2_total_counts:_738_Seed:_3_K:_25_length:_357	357
vOTU_151	1.94269	119038_2_total_counts:_534_Seed:_2_K:_25_length:_349	349
vOTU_118	1.88081	160_2_total_counts:_1030_Seed:_3_K:_25_length:_495	495
vOTU_147	1.875	60296_2_total_counts:_794_Seed:_2_K:_25_length:_368	368
vOTU_182	1.83113	58655_3_total_counts:_818_Seed:_4_K:_25_length:_302	302
vOTU_175	1.8109	95022_2_total_counts:_512_Seed:_2_K:_25_length:_312	312
vOTU_142	1.73138	33456_2_total_counts:_799_Seed:_4_K:_25_length:_376	376
vOTU_122	1.70753	62167_2_total_counts:_983_Seed:_3_K:_25_length:_465	465
vOTU_167	1.67081	4581_4_total_counts:_1133_Seed:_5_K:_25_length:_322	322
vOTU_85	1.59906	58070_3_total_counts:_2596_Seed:_4_K:_25_length:_848	848
vOTU_178	1.55882	151973_2_total_counts:_631_Seed:_5_K:_25_length:_306	306
vOTU_140	1.5582	61764_3_total_counts:_1169_Seed:_2_K:_25_length:_378	378
vOTU_136	1.54613	35477_3_total_counts:_1262_Seed:_4_K:_25_length:_401	401
vOTU_111	1.53093	116472_2_total_counts:_909_Seed:_2_K:_25_length:_582	582
vOTU_163	1.4893	34695_2_total_counts:_691_Seed:_2_K:_25_length:_327	327
vOTU_162	1.42813	150732_2_total_counts:_730_Seed:_4_K:_25_length:_327	327
vOTU_154	1.4058	64062_2_total_counts:_761_Seed:_2_K:_25_length:_345	345
vOTU_179	1.37255	155141_2_total_counts:_477_Seed:_2_K:_25_length:_306	306
vOTU_161	1.36254	63434_2_total_counts:_490_Seed:_2_K:_25_length:_331	331
vOTU_159	1.2929	120792_3_total_counts:_859_Seed:_3_K:_25_length:_338	338
vOTU_177	1.27362	155933_2_total_counts:_633_Seed:_4_K:_25_length:_307	307
vOTU_103	1.18069	149274_2_total_counts:_1187_Seed:_3_K:_25_length:_642	642
vOTU_143	1.17647	161001_2_total_counts:_637_Seed:_2_K:_25_length:_374	374
vOTU_130	1.1295	155663_2_total_counts:_740_Seed:_2_K:_25_length:_417	417
vOTU_171	1.06646	149082_2_total_counts:_673_Seed:_5_K:_25_length:_316	316
vOTU_129	0.990544	155087_2_total_counts:_779_Seed:_2_K:_25_length:_423	423
vOTU_150	0.845506	34480_2_total_counts:_589_Seed:_2_K:_25_length:_356	356
vOTU_110	0.843803	30645_5_total_counts:_2820_Seed:_4_K:_25_length:_589	589
vOTU_126	0.671111	92269_2_total_counts:_813_Seed:_3_K:_25_length:_450	450
vOTU_169	0.3875	154270_3_total_counts:_793_Seed:_4_K:_25_length:_320	320
vOTU_176	0.305466	5135_3_total_counts:_781_Seed:_6_K:_25_length:_311	311
vOTU_144	0.0537634	62821_2_total_counts:_663_Seed:_2_K:_25_length:_372	372

DNA metagenome

vOTU	coverage	contig_id	length
vOTU_22	3134.75	NODE_142_length_837_cov_2004.61	837
vOTU_33	2163.05	NODE_257_length_556_cov_1608.69	556
vOTU_17	2142.13	NODE_59_length_1601_cov_1305.56	1601
vOTU_11	1691.91	NODE_38_length_2005_cov_1102.3	2005
vOTU_43	1428.4	NODE_390_length_422_cov_1051.65	422
vOTU_2	41.9851	NODE_1_length_14405_cov_25.8403	14405
vOTU_13	28.2579	NODE_47_length_1772_cov_19.8427	1772
vOTU_27	21.6324	NODE_208_length_612_cov_13.7487	612
vOTU_3	19.1985	Bin2	11990
vOTU_19	15.2828	NODE_91_length_1174_cov_9.41555	1174
vOTU_1	11.518	Bin1	24637
vOTU_18	10.9875	NODE_86_length_1202_cov_8.1578	1202
vOTU_48	10.0293	NODE_488_length_376_cov_4.82866	376
vOTU_10	8.0813	NODE_31_length_2251_cov_3.51685	2251

vOTU	coverage	contig_id	length
vOTU_9	7.74051	NODE_26_length_2424_cov_5.68763	2424
vOTU_30	7.69665	NODE_241_length_567_cov_5.27539	567
vOTU_8	7.26818	NODE_18_length_2681_cov_5.18012	2681
vOTU_21	7.07198	NODE_130_length_903_cov_4.64741	903
vOTU_23	7.03329	NODE_164_length_751_cov_2.38075	751
vOTU_16	6.72788	NODE_55_length_1650_cov_4.20313	1650
vOTU_15	6.68272	NODE_54_length_1661_cov_6.05168	1661
vOTU_24	6.5057	NODE_177_length_702_cov_4.84853	702
vOTU_37	6.24462	NODE_306_length_511_cov_3.29825	511
vOTU_47	5.92708	NODE_470_length_384_cov_4.27964	384
vOTU_12	5.84069	NODE_40_length_1902_cov_3.04169	1902
vOTU_14	5.46983	NODE_50_length_1707_cov_4.08414	1707
vOTU_36	5.40571	NODE_291_length_525_cov_3.8	525
vOTU_49	4.824	NODE_491_length_375_cov_3.20938	375
vOTU_53	4.13772	NODE_654_length_334_cov_2.48746	334
vOTU_42	3.85681	NODE_373_length_433_cov_3.62169	433
vOTU_39	3.71257	NODE_313_length_501_cov_2.73543	501
vOTU_28	3.69454	NODE_229_length_586_cov_3.24482	586
vOTU_31	3.5922	NODE_245_length_564_cov_2.4165	564
vOTU_20	3.58394	NODE_97_length_1108_cov_2.96771	1108
vOTU_54	3.53614	NODE_664_length_332_cov_2.11913	332
vOTU_52	3.45906	NODE_620_length_342_cov_2.31359	342
vOTU_26	3.41479	NODE_204_length_622_cov_2.08289	622
vOTU_46	3.33938	NODE_466_length_386_cov_1.8429	386
vOTU_41	3.23502	NODE_371_length_434_cov_2.53298	434
vOTU_32	3.16014	NODE_249_length_562_cov_2.46351	562
vOTU_50	3.1532	NODE_547_length_359_cov_2.16118	359
vOTU_45	3.03	NODE_441_length_400_cov_1.93623	400
vOTU_40	2.79287	NODE_358_length_449_cov_1.63959	449
vOTU_35	2.5226	NODE_262_length_553_cov_1.93173	553
vOTU_38	2.47554	NODE_307_length_511_cov_1.53289	511
vOTU_25	2.25653	NODE_192_length_651_cov_2.01174	651
vOTU_55	2.25378	NODE_670_length_331_cov_1.9058	331
vOTU_34	2.217	NODE_261_length_553_cov_2.07229	553
vOTU_57	2.05363	NODE_738_length_317_cov_1.98092	317
vOTU_29	2.05181	NODE_235_length_579_cov_1.58779	579
vOTU_56	1.89815	NODE_702_length_324_cov_1.64312	324
vOTU_44	1.88078	NODE_418_length_411_cov_1.70506	411
vOTU_58	1.87025	NODE_741_length_316_cov_2.0613	316
vOTU_51	1.4661	NODE_574_length_354_cov_1.08027	354
vOTU_59	1.28025	NODE_755_length_314_cov_1.40541	314
